# The Importance of Positive Youth Development Attributes to Life Satisfaction and Hopelessness in Mainland Chinese Adolescents

**DOI:** 10.3389/fpsyg.2020.553313

**Published:** 2020-09-30

**Authors:** Zheng Zhou, Daniel T. L. Shek, Xiaoqin Zhu

**Affiliations:** ^1^Research Institute of Social Development, Southwestern University of Finance and Economics, Chengdu, China; ^2^Department of Applied Social Sciences, The Hong Kong Polytechnic University, Hong Kong, China

**Keywords:** positive youth development, life satisfaction, hopelessness, Chinese adolescents, psychological well-being

## Abstract

In contrast to mainstream theories focusing on adolescent developmental deficits, the positive youth development (PYD) approach highlights adolescent developmental plasticity and potentials. There are rich empirical research and review studies showing that PYD attributes promote adolescent well-being. However, the existing literature shows several limitations. First, while there are many Western studies, Chinese studies are sparse, particularly studies in mainland China. Second, most PYD studies are cross-sectional studies with data collected at one single time point. Third, researchers in different Chinese contexts seldom employed validated Chinese scales assessing different domains of PYD attributes. Fourth, few studies have examined the relationships between PYD attributes (measures of eudaimonic well-being) and life satisfaction (measure of hedonic well-being) and hopelessness (measure of negative well-being) in a single study. Finally, the role of life satisfaction or hopelessness as a mediator of the relationship between PYD attributes and psychological well-being is unclear. To explore the importance of PYD attributes to Chinese adolescent psychological well-being, we conducted a longitudinal study with two waves of data collected from junior high school students in mainland China (*N* = 2,648). At both times, validated measures of PYD attributes, life satisfaction, and hopelessness were employed. Results showed that PYD attributes concurrently and longitudinally predicted life satisfaction and hopelessness with small effect sizes. Cross-lagged panel analyses showed that while Wave 2 life satisfaction did not serve as a mediator of the relationship between Wave 1 PYD attributes and Wave 2 hopelessness, Wave 2 hopelessness served as a mediator of the link from Wave 1 PYD attributes to Wave 2 life satisfaction. In view of the paucity of research findings in this area, the present findings clarify the association between PYD attributes and hedonic well-being (indexed by life satisfaction) and negative psychological well-being (indexed by hopelessness) in Chinese adolescents.

## Introduction

With reference to the notion of “storm and stress” in adolescence and the Freudian view that turmoil is inevitable in adolescent development, adolescents have been seen as “problems to be fixed” and “troublesome” (Arnett, 1999; Buchanan and Hughes, 2011). However, in the past decades, there has been an appeal for a shift from the pathological orientation to positive growth orientation in adolescent development, with the positive youth development (PYD) as one of the positive approaches (Tolan et al., 2016).

With specific reference to PYD models, different researchers have used the term “positive youth development” differently with several models associated with the term. Benson et al. (2006) outlined three theoretical foundations underlying the PYD approach, including theories of human development, frameworks on contextual influence, and models on community influence and change. Besides, different principles are upheld in PYD frameworks, such as believing that every young person has the inborn capacity to grow and develop (Hamilton et al., 2004; Lerner et al., 2015). In their review of the conceptual frameworks in the field of PYD, Shek et al. (2019a) identified several PYD models that are commonly used. The first approach is the developmental assets model. For example, Benson et al. (2011) proposed that 20 external assets and 20 internal assets are critical to the positive development of adolescents. While external assets refer to the positive experience derived from other people and the external world (“support,” “empowerment,” “boundaries and expectations,” and “constructive use of time”) which promote adolescent development, internal assets are individual qualities (“commitment to learning,” “positive values,” “social competencies,” and “positive identity”) that guide positive choices leading to thriving. The second approach is the model emphasizing 5Cs/6Cs (connection, competence, confidence, character, care, and contribution) (Lerner et al., 2011). The third approach is “social and emotional learning” (SEL) that highlights the importance of psychosocial skills related to oneself (“self-awareness,” “self-management,” and “responsible decision making”) and others (“social awareness” and “relationship skills”). The fourth approach is the “being” perspective where optimal youth development is seen as a function of “being” (e.g., character, spirituality, and inner strengths) rather than “doing” (e.g., skills and achievements).

The final PYD approach is an inductive framework derived from a review of PYD programs. Catalano et al. (2004) reviewed 77 PYD programs and concluded that 15 PYD constructs could be identified from programs showing positive outcomes. These PYD attributes include having good relationships with positive peers and adults (bonding), competence to overcome adversity (resilience), ability to think logically, creatively, and critically (cognitive competence), ability to manage emotions (emotional competence), ability to maintain good social relationships (social competence), ability to take verbal and non-verbal actions (behavioral competence), ability to make sound moral judgment (moral competence), ability to take age-appropriate action by oneself (self-determination), belief that one has abilities and is able to attain goals (self-efficacy), having positive self-perceptions (positive and healthy self-identity), having good relationship with oneself, others, and higher being (spirituality), optimism (belief in the future), developing prosocial behavior (promotion of prosocial norms), engagement in prosocial behavior (prosocial involvement), and appreciation of young people’s positive behavior (i.e., desirable behavior of young people is properly recognized).

We used Catalano et al.’s (2004) framework to define PYD attributes in this study with three justifications. First, Catalano et al. (2004) inductively derived these PYD attributes from effective PYD programs, hence underscoring their conceptual and applied importance. Second, these PYD attributes integrate the PYD concepts in different models. For example, PYD attributes of bonding, psychosocial competence, and positive and healthy identity in this model are consistent with some of the internal and external assets in the developmental assets modes. Similarly, PYD attributes of psychosocial competence, self-determination, and prosocial involvement are in line with the 5Cs/6Cs models. Furthermore, the focus on cognitive, social, emotional, behavioral, and moral competences in the PYD attributes echoes the assertions of the Social-Emotional Learning framework. The final justification is that researchers have developed the Chinese Positive Youth Development Scale (CPYDS) to assess different aspects of PYD attributes (Shek et al., 2007; Shek and Ma, 2010) in the Chinese context. The use of the framework of Catalano et al. (2004) enables us to use the CPYDS to measure Chinese adolescents’ PYD attributes in the present study.

As the present study examined the relationship between PYD attributes covered in Catalano et al.’s (2004) model and psychological well-being indexed by life satisfaction and hopelessness, clarification of the concept of psychological well-being is in order. Dodge et al. (2012) pointed out that the concept of psychological well-being is complex and there are many definitions. According to Ryff and Keyes (1995), psychological well-being can be understood in terms of two perspectives, including psychological ill-being (or negative psychological well-being) and positive psychological well-being. In mainstream psychiatry and clinical psychology, the concept of psychological well-being has traditionally been conceived in terms of the absence of symptoms and dysfunction (Joseph and Wood, 2010), such as those psychological symptoms described in the 5th edition of the Diagnostic and Statistical Manual for Mental Disorders (American Psychiatric Association, 2013). It is noteworthy that psychological ill-being is still the focus of many clinical disciplines where the goal of intervention is to reduce or minimize psychological symptoms. One common symptom that occurs in different mental disorders is the loss of hope (i.e., hopelessness). In particular, adolescents have expectations and aspirations about the future (e.g., getting admitted to a university or getting married). When an adolescent is hopeless, he/she expects a dark future.

In this study, we focused on hopelessness as an indicator of negative psychological well-being for two reasons. First, hopelessness can be regarded as an adolescent internalizing problem. Conceptually speaking, internalizing problems are regarded as “the subgroup of psychopathology that involves disturbances in emotion or mood” (Graber, 2004, p. 587) with high negativity directed inward which include anxiety, depression, and suicide (Liu et al., 2011). As the feeling of hopelessness is an integral part of adolescent depression and suicide, it can be regarded as a form of internalizing problem. Second, there is an increase in hopelessness symptoms in the adolescent years (Shek and Liang, 2018). Although one may argue that hope (optimism or beliefs in the future) as a PYD attribute is conceptually similar to hopelessness, research showed that they are conceptually different. For example, based on the responses of 2,106 respondents, Huen et al. (2015) showed that these two constructs are related but distinct. Besides, studies have also treated PYD attributes (e.g., resilience) and hopelessness separately (Hjemdal et al., 2012).

While the absence of symptoms or dysfunction is central to the perspective of negative psychological well-being, Ryff and Singer (1996) argued that “absence of illness” is not equivalent to “presence of wellness” (p. 14). Joseph and Wood (2010) also pointed out that “studies of positive psychological functioning have been far outweighed by those concerned with psychological distress and dysfunction” (p. 830). As such, there is a call to conceive psychological well-being in terms of positive functioning and experience. According to Ryan and Deci (2001), there are two general approaches on psychological well-being regarding optimal experience, which include the hedonic approach “which focuses on happiness and defines well-being in terms of pleasure attainment and pain avoidance” and the eudaimonic approach “which focuses on meaning and self-realization and defines well-being in terms of the degree to which a person is fully functioning” (p. 141). For the “hedonic” conception of psychological well-being, researchers have defined it as “subjective well-being” which includes the presence of pleasurable experience, enjoyment, satisfaction, and comfort (Huta and Waterman, 2014). In the present study, we focused on life satisfaction (i.e., overall cognitive evaluation of one’s life quality, Gilman and Huebner, 2003) as an indicator of hedonic well-being (subjective well-being) which is regarded as a consequence of PYD attributes (Martela and Sheldon, 2019). In this study, we used life satisfaction as an indicator of subjective well-being for two reasons. First, according to Gallagher et al. (2009), hedonic conceptions of well-being (such as life satisfaction) have been widely studied as indicators of psychological well-being. Second, there has been a downward trend in life satisfaction during adolescence (e.g., Shek and Liang, 2018).

With reference to the above conceptions of psychological well-being, one might ask whether the PYD attributes in Catalano et al. (2004) are also measures of psychological well-being. There are four points one should note. First, there are different aspects of psychological well-being (Dodge et al., 2012). Theoretically, components of PYD have been regarded as indicators of eudaimonic well-being. In a review of the concept of eudaimonic well-being, Martela and Sheldon (2019) pointed out that there are 45 conceptions and 63 elements involved. From this review, the 15 PYD constructs in this study can be regarded as elements of eudaimonic well-being, with corresponding elements in Martela and Sheldon’s (2019) framework shown in the brackets: resilience (resilience), bonding (social support), cognitive competence (clear thinking), emotional competence (positive emotion/competence), social competence (competence), behavioral competence (personal expressiveness), moral competence (being a good person), self-determination (self-determination), self-efficacy (efficacy), positive identity (self-acceptance and self-esteem), spirituality (meaning in life), beliefs in the future (optimism), prosocial norms (being a good person), prosocial involvement (prosocial impact), and recognition for positive behavior (accomplishment). Second, while PYD can be understood as eudaimonic well-being elements, we have to take this view in a cautious manner because “there is no consensus about what the key elements or sets of elements are” (Martela and Sheldon, 2019, p. 459). Third, there are theories differentiating PYD attributes, hopelessness, and subjective well-being as separate but related constructs. For example, in a review of the determinants of hedonic well-being, Lent (2004) proposed that PYD attributes (e.g., self-efficacy and optimism) influence global life satisfaction (hedonic well-being). Finally, there are studies treating PYD attributes, hopelessness, and life satisfaction as separate constructs. For example, Mohamad et al. (2014) examined the influence of PYD attributes on life satisfaction. Similarly, Hjemdal et al. (2012) examined the impact of resilience (a PYD attribute) on hopelessness. Other researchers also considered PYD constructs as a predictor of life satisfaction (e.g., Lee et al., 2012; Di Fabio and Palazzeschi, 2015).

Theoretically, at least two theoretical accounts propose that PYD attributes would positively influence life satisfaction and negatively influence hopelessness. First, in different PYD models, theorists asserted that as developmental assets, PYD attributes promote adolescent development. For example, Hamilton et al. (2004) suggested that psychological and emotional assets (PYD attributes) facilitate positive adolescent development, such as leading to good mental health and planning for the future. Benson et al. (2006) also proposed that “presence of high levels of developmental assets results *over time* in (a) lessened risk behaviors; (b) increased academic achievement; (c) increased contribution; and (d) higher levels of other thriving indicators” (p. 894). Martela and Sheldon (2019) also proposed that eudaimonic well-being (such as purpose in life and optimism) influences subjective well-being (such as life satisfaction). These theoretical accounts provide the basis of the hypothesis that PYD is associated with life satisfaction and hopelessness.

Empirically, research findings show a negative relationship between PYD attributes and psychological ill-being indicated by psychological symptoms: Dupéré et al. (2012) showed that self-efficacy was negatively related to internalizing behavior in adolescents; Davis and Humphrey (2012) showed that emotional intelligence predicted depression and other internalizing behavior. Existing research has also revealed that different aspects of PYD including future orientation (Hamilton et al., 2015), school engagement (Cammarota, 2011), and positive thinking (Ciarrochi and Heaven, 2008; Brandon and Lauren, 2010) were negatively related to hopelessness. In a recent study, Zhou et al. (2020) showed that PYD predicted adolescent depression (where hopelessness is an integral component) over time. However, it is noteworthy that research on the relationship between PYD attributes and adolescent hopelessness is very limited.

On the other hand, evidence on the positive relationship between PYD attributes and positive well-being has also been found. For example, Ciarrochi and Scott (2006) showed a significant relationship between emotional management and well-being defined in terms of emotional health. Di Fabio and Palazzeschi (2015) showed positive relationships between resilience and life satisfaction. For prosocial behavior as a PYD attribute, Aknin et al. (2013) conducted a survey in 136 countries and found that prosocial behavior was associated with greater happiness, which was consistent across countries with different cultural backgrounds and economic status. Dou et al. (2019) also showed that prosocial behavior predicted adolescent life satisfaction. Moreover, life satisfaction was shown to be related to hope (Gilman et al., 2006), self-efficacy (Lent, 2004), and emotional intelligence (Sánchez-Álvarez et al., 2015). Valois (2014) also reported that developmental assets were positively related to life satisfaction. Nevertheless, he commented that related studies in the field “have been scarce” (p. 3586) and further argued that “more research is needed in regard to how developmental assets are related to emotional and social well-being in adolescence” (p. 3587).

In short, theoretical accounts and research findings generally suggest that PYD attributes defined in terms of the model of Catalano et al. (2004) are positively related to positive psychological well-being but negatively related to negative psychological well-being. Unfortunately, empirical studies on these theoretical expectations are few. For example, Schultze-Lutter et al. (2016) reported in a review study that there were only six studies on the relationship between resilience as a PYD indicator and well-being. There are also very few studies that examined the relationships between PYD attributes and both hedonic well-being and psychological ill-being in a single study.

On top of the possible influence of PYD on subjective well-being and psychological ill-health, how PYD attributes were associated with one psychological well-being measure (e.g., hopelessness) via another well-being mediator (e.g., life satisfaction) is far from clear. Based on the limited literature, two non-mutually exclusive expectations can be formed. The first expectation is that life satisfaction is a mediator in the relationship between PYD attributes and negative psychological well-being. This expectation is supported by some studies: Suldo and Huebner (2004) reported that parenting (bonding) influenced problem behavior via satisfaction with life; Yamawaki et al. (2011) showed that life satisfaction served as a mediator in the association between parental bonding and general psychological morbidity; Lopez-Zafra et al. (2019) showed that social support and emotional intelligence influenced psychological symptoms with life satisfaction as a mediator; Sun and Shek (2010, 2012) showed that life satisfaction was a mediator of the influence of PYD attributes on adolescent problem behavior. Based on 6 waves of data, Shek and Chai (2020) also reported that PYD predicted academic stress with the mediation of academic satisfaction.

On the other hand, studies show that negative psychological well-being influences life satisfaction. For example, Sánchez-Álvarez et al. (2015) showed that negative affect mediated the influence of emotional intelligence on life satisfaction. Hopelessness also mediated the influence of belief in a just world (Ucar et al., 2019) and bicultural stress (Piña-Watson et al., 2015) on life satisfaction. Hopelessness denotes a set of “negative cognitive schemas” which include “negative expectations about the future” (Ucar et al., 2019, p. 69). With these negative cognitive schemas, hopeless individuals would evaluate their experiences and general life more negatively, which leads to their decreased life satisfaction. Hence, the second hypothesis is that negative psychological well-being indexed by hopelessness would mediate the link from PYD attributes to life satisfaction. It is noteworthy that there is no known scientific research examining the second hypothesis.

There are also several methodological limitations of the studies in this field. First, although pretest and posttest data are commonly collected in PYD intervention studies to demonstrate the benefits of promoting PYD attributes (e.g., Lin and Shek, 2019), comparatively fewer longitudinal survey studies have been conducted to look at the relationship between PYD attributes and positive as well as negative psychological well-being. Methodologically, longitudinal studies can give more definitive answers to the causal relationships between predictors and developmental outcomes (Farrington, 1991). Second, PYD studies have been predominantly conducted in Western societies (Wiium and Dimitrova, 2019). As Benson et al. (2006) argued, the role of developmental assets may work differently in different cultural contexts. Hence, collecting data from non-Western contexts is important. Besides, as predictors of life satisfaction varied across cultures (Oishi et al., 2009; Suh and Choi, 2018) and there are cross-cultural differences in subjective well-being (Tov and Nai, 2018), studies on the inter-relationships between PYD attributes and life satisfaction as well as hopelessness in non-Western contexts, would be theoretically illuminating. Third, as PYD is a multi-dimensional construct (e.g., the 40 developmental assets and the 15 PYD attributes), there is a need to use validated PYD assessment tools to assess these PYD domains such as psychosocial competence and positive identity. Unfortunately, most of the existing PYD tools were developed in the West (Shek et al., 2007).

In this study, we examined the association between PYD attributes and psychological well-being amongst Chinese adolescents in mainland China with several justifications. First, psychological well-being is found to be deeply influenced by culture (Kitayama and Markus, 2000; Suh and Choi, 2018). However, the number of related studies in mainland China is small. Utilizing “positive youth development and life satisfaction” as the search term, there were 410 results in PsycINFO in June 2020. However, when we used the search term “positive youth development and life satisfaction and China,” there were only 22 results. Besides, while there were 51 citations for “positive youth development and hopelessness,” there was no study under “positive youth development and hopelessness and China.” Finally, while the search term “life satisfaction and hopelessness” led to 322 results, adding “China” to the search term resulted in only 16 citations.

The second justification is the large size of the adolescent population in China. In 2018, the population of adolescents aged 10–19 in China was 166.857 million while the number of adolescents of the same age range in the world was 1,236.747 million (UNICEF, 2019). Thus, Chinese adolescents constituted roughly 13.5% of the world adolescent population, which suggests a strong need to examine whether Western PYD theories and findings can be generalized to Chinese adolescents (i.e., generalizability across populations).

The third justification is that there are differences and similarities in cultural emphases between Western and Chinese cultures regarding child and adolescent development. In terms of differences, some PYD constructs closely related to individualism and the autonomous self, such as self-determination, self-efficacy, and positive identity, were not emphasized in the traditional Chinese culture. For example, as relationship harmony is more stressed in cultures with a high level of interdependence such as China (Lu et al., 2001; Uchida and Kitayama, 2009; Hitokoto and Uchida, 2015; Shin et al., 2018), bonding and prosocial norms might be more significant in predicting well-being among Chinese adolescents than their counterparts in the West. Besides, hedonistic goals and ideals in well-being were not promoted in traditional Chinese social philosophies (Zhang and Veenhoven, 2008). For example, Confucianism focuses on the pursuit of self-cultivation in the moral and virtuous domains; Buddhism and Taoism take the stand that pursuit of hedonistic pleasure should not be the goal of human life. Yang and Zhou (2017) also pointed out that individual emotion is not fundamental to well-being in Chinese people. Empirically, while there was no difference between American and Chinese young adults on resilience capability (Zheng et al., 2020), Church et al. (2012) showed that Asian colleague students’ autonomy and self-acceptance were significantly lower than their counterparts in the United States. Liu et al. (2016) also reported that Chinese adolescents showed higher modesty, self-regulation, prudence than curiosity and humor (Liu et al., 2016), whereas Western adolescents scored higher on integrity, kindness, gratitude but lower on modesty and self-regulation (Park and Peterson, 2006).

On the other hand, there are some similarities between Western and Chinese cultures regarding PYD attributes. PYD constructs such as resilience, moral competence, and spirituality validated in the western population were also strongly emphasized in the traditional Chinese culture. For example, the Chinese belief of “*gu tian jiang da ren yu si ren ye, bi xian ku qi xin zhi, lao qi jin gu, e qi ti fu”* (“when Heaven is about to confer a great office on any man, it first exercises his mind with suffering, and his sinews and bones with toil. It exposes his body to hunger”) underscores the importance of resilience (Chinese Text Project, 2006a). The Chinese belief of “*xiu shen, qi jia, zhi guo ping tian xia”* (“the ancients who wished to illustrate illustrious virtue throughout the kingdom, first ordered well their states. Wishing to order well their states, they first regulated their families. Wishing to regulate their families, they first cultivated themselves”) also highlights the importance of moral competence and bonding (Chinese Text Project, 2006b). In their examination of how children were raised in the traditional Chinese society with reference to “*Di Zi Gui*” (Standards for being a good student and child), Shek and Law (2019) concluded that while some of the traditional emphases (such as moral and virtue development, strong family support, maintain good social relationships with others, observing rules and norms, and helping others) are consistent with the contemporary emphases in PYD literature, some PYD attributes such as self-determination, self-efficacy, and emotional competence are not emphasized in “*Di Zi Gui.*” In addition, Chinese people have become more “Westernized” with the process of globalization. For example, Steele and Lynch (2013) concluded that Chinese society has been increasingly adopting individualistic values. Moreover, Zhu and Shek (2020) showed that PYD program based on Catanalo’s framework of PYD constructs could be successfully implemented in middle schools in China (Zhu and Shek, 2020).

To sum up, these cultural differences and similarities would motivate psychologists to ask whether the claims of Western PYD models (e.g., PYD attributes would be positively related to psychological well-being) and related findings would hold in the Chinese context.

The above three justifications support the importance of conducting this study in Chinese adolescents. In the realm of science, under the principle of “approximation and accumulation of truth,” replication (irrespective of whether there are contextual differences across studies) is an important criterion to determine the validity of research findings. In the field of psychological research, in addition to the warning that “replicability in research is an important component of cumulative science…yet relatively few close replication attempts are reported in psychology” (p. 217), Brandt et al. (2014) actually developed a “replication recipe” guiding researchers.

With reference to the above research gaps in the literature, we attempted to examine three research questions using data from a 2-wave longitudinal study as follows:

*Research Question 1*: Do PYD attributes predict life satisfaction? Based on the literature, we expected that PYD attributes would have positive concurrent and longitudinal prediction of life satisfaction (Hypothesis 1).

*Research Question 2*: Do PYD attributes predict hopelessness? Based on the preceding discussion, we hypothesized that PYD attributes would negatively predict adolescent hopelessness at each wave and over time (Hypothesis 2).

*Research Question 3*: What are the inter-relationships amongst PYD attributes, life satisfaction, and hopelessness? With reference to the preceding discussion, there are two mutually non-exclusive hypotheses for this research question. First, based on previous studies (e.g., Sun and Shek, 2010, 2012), it was hypothesized that life satisfaction would mediate the link from PYD attributes to hopelessness. Second, as studies have also shown that psychological symptoms predicted life satisfaction (e.g., Fergusson et al., 2015), it was expected that hopelessness would serve as a mediator of the relationship between PYD attributes and life satisfaction.

To answer the above research questions, a longitudinal study was conducted with two waves of data collected over 2 years. With an interval of only 1 year, this study can be regarded as a “short-term” longitudinal study. As early Chinese adolescents encounter many adjustment problems (Shek and Lin, 2017), it would be interesting to ask whether PYD attributes predict student well-being from Grade 7 to Grade 8. Despite their limitations, short-term longitudinal studies with two waves of data collected over a short period of time are not uncommon in the field. For example, Yao and Zhong (2014) conducted a two-wave longitudinal study (*N* = 361) with an interval of 4 months to examine the relationships amongst loneliness, contact with friends, and Internet addiction symptoms. Konze et al. (2017) collected two waves of data from 139 employees with a time lag of 6 months to examine workload and emotional exhaustion.

Consistent with the other studies in the field (e.g., Shek et al., 2018, 2019b), we treated age, gender, and family intactness as covariates. Previous studies showed that demographic variables such as age and gender might have a weak relationship with adolescent well-being (Proctor et al., 2009). For example, there was a general decline in life satisfaction and an increasing trend in hopelessness over time (Park, 2005; Shek and Liang, 2018). For gender, while some studies reported higher levels of well-being among boys, some reported higher well-being in girls, and some other studied showed no gender differences in well-being (see Batz and Tay, 2018 for a review). For family intactness, adolescents in intact families tended to have better development and higher levels of well-being (Antaramian et al., 2008; Shek and Liang, 2018).

## Materials and Methods

### Participants and Procedures

In August 2016, four junior secondary schools in mainland China were invited to participate in a 2-wave study on Chinese adolescents’ adjustment and well-being. These participation schools were located in different cities including Suzhou (in Jiangsu Province), Jiujiang (in Jiangxi Province), Shanwei (in Guangdong Province), and Zhaoqing (in Guangdong Province). These four schools were all public secondary schools. At the beginning of the school year of 2016/2017, a total of 1,362 Grade 7 and 1,648 Grade 8 students in these four participating schools completed a survey measuring adolescent psychosocial competence, problem behavior, and well-being. Among these students, 1,305 and 1,343 at the two grade levels, respectively, responded to the same questionnaires after 1 year. Thus, the overall attrition rate was 4.19 and 18.51% among Grade 7 and Grade 8 students, respectively.

Among the matched sample (*N* = 2,648), 1,513 were males, 1,109 were females, and the remaining 26 students did not report information on their gender. The mean age of the participants in the matched sample was 13.12 ± 0.81 years old at Wave 1. For family intactness, 2,225 students were from intact families (i.e., their parents were married for the first time). The other 401 students reported that their parents were separated, divorced, or re-married (i.e., staying in non-intact families).

The study was approved by the “Human Subjects Ethics Subcommittee” at the corresponding author’s institution. Before the commencement of the study, the students were fully explained about the study objectives as well as the confidential and anonymous nature of the data collected. Prior to students’ participation, consent was obtained from the key stakeholders, including school administrators, participating students, and parents of the students.

### Measures

**FIGURE 1:**
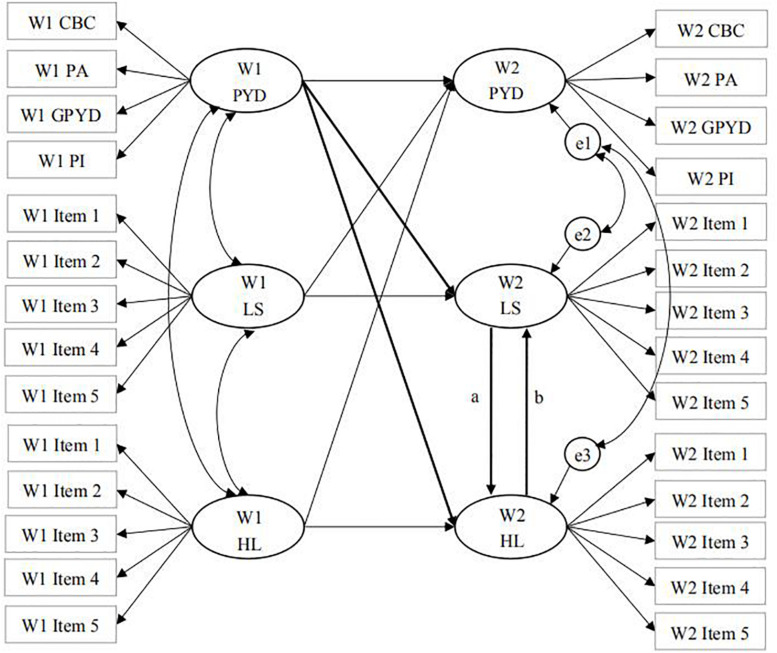
Conceptual paradigms of cross-lagged mediation effect models. The model with path a indicates the mediation effect of life satisfaction and the model with path b indicates the mediation effect of hopelessness. The two models were separately evaluated. WI, Wave 1; W2, Wave 2; PYD, positive youth development quality; LS, life satisfaction; HL, hopelessness; CBC, cognitive-behavioral competence; PA, prosocial attributes; GPYD, general positive youth development; PI, positive identity; e1, residual of Wave 2 PYD; e2, residual of Wave 2 LS; e3, residual of Wave 2 HL.

A questionnaire containing various measures of the psychosocial adjustment of adolescents was used. In the questionnaire, validated measures of PYD attributes, life satisfaction, hopelessness, depression, Internet addiction, delinquency, suicidal behavior, materialism, egocentrism, empathy, academic achievement, and academic anxiety (e.g., Shek and Liang, 2018; Shek et al., 2018) were used. In this study, we focused on how PYD attributes are linked to life satisfaction (hedonic well-being) and hopelessness (psychological ill-being). There are three justifications to focus on PYD and well-being in this study. First, the study was guided by well-articulated research questions and conceptual models (see Figure 1). Second, the inclusion of too many variables in a single study would dilute the focus of the study. Third, for datasets involving multiple measures, it is not uncommon for researchers to focus on different research questions in different journal articles (e.g., Lyons et al., 2014, 2016; Koning et al., 2018; van Den Eijnden et al., 2018).

#### PYD Attributes

We used the 80-item “Chinese Positive Youth Development Scale (CPYDS)” to measure PYD qualities. With reference to the 15 PYD constructs highlighted in the effective PYD programs (Catalano et al., 2004), this scale assessed Chinese adolescents’ PYD attributes. Previous studies showed that this scale possessed very good psychometric properties, including reliability, criterion-related validity, and construct validity (Shek et al., 2007; Shek and Ma, 2010). Besides 15 subscales based on the 15 primary factors on PYD attributes, there were four higher-order factors. First, “Cognitive-behavioral competence” higher-order factor includes three subscales, including “cognitive competence,” “behavioral competence,” and “self-determination.” Second, “Prosocial attributes” higher-order factor covers two subscales, including “prosocial norms” and “prosocial involvement.” Third, “General PYD qualities” high-order factor contains eight subscales (i.e., “bonding,” “resilience,” “social competence,” “recognition for positive behavior,” “emotional competence,” “moral competence,” “self-efficacy,” and “spirituality” subscales). Finally, “Positive identity” higher-order factor includes “clear and positive identity” and “beliefs in the future” subscales. This validated measure of PYD is important for survey research as far as operationalization is concerned. The dimensions of PYD can also be used as observed measures to represent the latent construct of PYD. For all items in the CPYDS, a 6-point scale (“1 = strongly disagree”; “6 = strongly agree”) was used for all items. For scale scores of the primary factors, higher-order factors, and the whole PYD scale (i.e., mean total PYD scores), the average scores of the related items were computed. All measures in this study showed sufficient internal consistency (see Table 1). Although the reliability of self-efficacy subscale was not high, it is not unacceptable because there were only two items and the mean item-total correlation was not low.

**TABLE 1:** Descriptive statistics and reliability measures.

Measures	Wave 1				Wave 2			
	Mean	*SD*	*α*	Mean inter-item correlation	Mean	*SD*	*α*	Mean inter-item correlation
CBC	4.82	0.74	0.91	0.38	4.95	0.78	0.94	0.49
PA	4.87	0.84	0.86	0.40	5.01	0.85	0.90	0.48
PI	4.73	1.00	0.88	0.44	4.87	1.02	0.90	0.49
GPYD	4.81	0.71	0.95	0.30	4.92	0.76	0.96	0.37
TPYD	4.78	0.69	0.97	0.31	4.90	0.73	0.98	0.38
LS	4.06	1.12	0.81	0.48	4.06	1.13	0.84	0.53
HL	2.76	1.19	0.78	0.41	2.74	1.27	0.82	0.47

*CBC, cognitive-behavioral competence; PI, positive identity; PA, prosocial attributes; GPYD, general positive youth development qualities; TPYD, total positive youth development qualities; LS, life satisfaction; HL, hopelessness.*

#### Life Satisfaction (LS)

The Chinese translated “Satisfaction with Life Scale” (Diener et al., 1985) which has been widely utilized in measuring Chinese people’s global life satisfaction (Shek and Li, 2016; Shek and Liang, 2018) was used. A 6-point rating scale (“1 = strongly disagree”; “6 = strongly agree”) was used to assess the participants’ perceived satisfaction with their life, such as whether they regarded their life condition as excellent. The internal consistency of the scale in the two waves was very good (Cronbach’s α = 0.81 and 0.84, respectively).

#### Hopelessness

A five-item “Chinese Hopelessness Scale” (Shek, 1993) was used. This scale was modified based on Beck et al.’s (1974) Hopelessness Scale. We used a six-point scale (“1 = strongly disagree,” “6 = strongly agree”) to assess the degree of hopelessness in the participants, such as whether they saw their future as gloomy. The values of Cronbach’s α were 0.78 and 0.82 at the two assessment occasions, respectively (see Table 1).

#### Covariates

As age, gender, and family intactness were covariates in the study, information on these variables was collected. For family intactness, those students who reported their parents were in the first marriage were considered living in intact families. Other students who reported that their parents were not in the first marriage (e.g., separated, divorced, or re-married) were considered living in non-intact families.

### Data Analyses

We used two strategies to answer the research questions in this study. The first strategy was to perform hierarchical multiple regression analyses based on ordinary least square (OLS) approach to examine the prediction of a PYD variable (e.g., positive identity composite scores) on an outcome (e.g., life satisfaction), with the predictors entered in blocks (e.g., control variables in Block 1 and Wave 1 PYD variable in Block 2). In the literature on longitudinal studies, researchers have commonly used this approach (Steinberg et al., 1989; Davis et al., 2019; Lázaro-Visa et al., 2019). We used SPSS 25.0 to conduct the multiple regression analyses as well as attrition and reliability analyses.

The second approach is to conduct cross-lagged path models based on structural equation modeling. Despite the overlap between OLS hierarchical multiple regression and structural equation modeling (Gefen et al., 2000), the latter has greater capability to deal with complex conceptual models (e.g., model with multiple pathways), different variables (e.g., latent variables), and measurement error. To understand how PYD attributes are related to life satisfaction and hopelessness (i.e., Research Question 3), two cross-lagged panel models were conducted to test the two models (see Figure 1) using AMOS 25.0. In the cross-lagged models, the longitudinal associations among target variables can be checked while the temporal stability of each variable between adjacent two assessment occasions and contemporaneous correlations between the variables are statistically controlled. Such an analytical technique was used in previous research to explore the longitudinal influence and mediation effect simultaneously based on 2-wave panel data (Tovel et al., 2019). In these two models, PYD attributes, life satisfaction, and hopelessness were all latent variables. PYD was indicated by the four higher-order factors calculated from CPYDS and life satisfaction and hopelessness were indicated by the respective five measuring items. Following the previous practice (Tovel et al., 2019), as we linked the cross-sectional association between life satisfaction and hopelessness at Wave 2 to test the mediation effect, we did not examine the longitudinal associations between life satisfaction and hopelessness over time.

As the two models testing the mediation effect of life satisfaction and hopelessness, respectively, were not nested models, “Akaike Information Criterion” (AIC) was used to compare model fit. As suggested by Burnham and Anderson (2002), the difference in the AIC between two models reliably informs the superiority of the fitting model with a lower AIC indicating a better-fitted model. While a difference less than 2 in AIC indicates minimal model fit differences between two models, a difference of 4–7 can adequately indicate that the model with lower AIC better fits the data. Generally speaking, the bigger the AIC difference, the better the model fit of the model with lower AIC as compared to the model with higher AIC. The model fit of the final selected model was assessed by following indices: “Comparative Fit Index” (CFI), “Goodness of Fit Index” (GFI), “Non-Normed Fit Index” (NNFI), and “Root Mean Square Error of Approximation” (RMSEA). A criterion for an adequate model fit was a value over 0.90 for CFI, GFI, and NNFI together with a value lower than 0.08 for RMSEA (Kline, 2015). Besides, the indirect effect was tested using bias-corrected (BC) bootstrapping (10,000 samples) with 95% BC confidence interval (CI) calculated (Preacher and Hayes, 2008). In both hierarchical multiple regression analyses and cross-lagged path models, control variables (age, gender, and family intactness) were considered in the related statistical models.

## Results

### Attrition Analyses

The descriptive statistics and reliability measures are presented in Table 1. Comparisons between the matched sample and those dropouts after the first wave of data collection revealed that there were no differences in the background demographic attributes, except for more dropouts in Grade 8 students living with their fathers than did the non-dropouts (90.39% versus 84.86%; χ^2^ = 5.84, *p* < 0.05, *φ* = 0.06). Regarding the differences between the two groups on PYD measures, life satisfaction, and hopelessness, results showed that dropouts in Grade 8 students displayed higher scores than did non-dropouts on the mean total PYD scores (4.89 versus 4.74; *t* = 3.55, *p* < 0.001, Cohen’s *d* = 0.23) and life satisfaction scores (4.59 versus 4.30; *t* = 4.59, *p* < 0.001, Cohen’s *d* = 0.29). As the number of dropouts was small and the differences between the dropout and non-dropout groups were not high, it can be concluded that sample attrition was not a major problem of the study.

### Prediction of PYD Attributes on Psychological Well-Being

In Table 2, PYD measures showed significant positive correlation with life satisfaction at each wave and across time after Bonferroni-correction (*p* = 0.05/15 = 0.003). PYD measures also showed a significant negative correlation with hopelessness at each wave and across time after Bonferroni-correction (*p* = 0.05/15 = 0.005). Cross-sectional regression analyses showed that after controlling the covariates (age, gender, family intactness), all PYD attributes positively predicted life satisfaction (*β* ranged between 0.44 and 0.60, *p*s < 0.001, Cohen’s *f*^2^ ranged between 0.24 and 0.56) and negatively predicted hopelessness (*β* ranged between –0.19 and –0.09, *p*s < 0.001, Cohen’s *f*^2^ ranged between 0.01 and 0.04) (Table 3). Hierarchical multiple regression analyses further showed that after removing the effects of the covariates, separate PYD measures at Wave 1 had positive predictive effects on Wave 2 life satisfaction (*β* ranged between 0.04 and 0.27, *p*s < 0.001, Cohen’s *f*^2^ ranged between 0.02 and 0.08) and negative predictive effects on Wave 2 hopelessness (*β* ranged between –0.23 and –0.15, *p*s < 0.001, Cohen’s *f*^2^ ranged between 0.02 and 0.05) (Table 4). Furthermore, hierarchical multiple regression analyses were conducted to examine the prediction of PYD attributes on the change in life satisfaction or hopelessness over time. After removing the effect of the covariates and Wave 1 criterion measure, results showed that Wave 1 “general PYD factor” and the mean total PYD predicted an increase in Wave 2 life satisfaction (*β* = 0.06 and 0.07, *p*s < 0.05, Cohen’s *f^2^* = 0.003) and all Wave 1 PYD measures predicted a decrease in hopelessness across time (*β* ranged between –0.19 and –0.12, *p*s < 0.001, Cohen’s *f*^2^ ranged between 0.02 and 0.04), with small effect sizes. As one PYD measure was included in each multiple regression analysis, there was no problem in multi-collinearity in the PYD predictors. In summary, the concurrent and longitudinal multiple regression analyses give initial support to Hypotheses 1 and 2.

**TABLE 2:** Correlation matrix.

Measures	Correlations												
	1	2	3	4	5	6	7	8	9	10	11	12	13
**Wave 1**													
1. CBC	–												
2. PA	0.66***	–											
3. PI	0.63***	0.92***	–										
4. GPYD	0.83***	0.71***	0.68***	–									
5. TPYD	0.90***	0.79***	0.76***	0.97***	–								
6. LS	0.51***	0.47***	0.48***	0.60***	0.62***	–							
7. HL	−0.07***	−0.11***	−0.10***	−0.13***	−0.13***	0.06***	–						
Wave 2													
8. CBC	0.44***	0.35***	0.35***	0.43***	0.46***	0.31***	−0.08***	–					
9. PA	0.34***	0.42***	0.40***	0.39***	0.42***	0.28***	−0.13***	0.66***	–				
10. PI	0.32***	0.39***	0.40***	0.37***	0.39***	0.29***	−0.12***	0.61***	0.94***	–			
11. GPYD	0.43***	0.38***	0.39***	0.49***	0.50***	0.38***	−0.12***	0.84***	0.69***	0.66***	–		
12. TPYD	0.45***	0.41***	0.40***	0.50***	0.51***	0.38***	−0.13***	0.91***	0.78***	0.74***	0.97***	–	
13. LS	0.21***	0.18***	0.20***	0.27***	0.27***	0.38***	−0.05***	0.44***	0.44***	0.46***	0.57***	0.58***	–
14. HL	−0.18***	−0.19***	−0.16***	−0.22***	−0.23***	−0.10***	0.33***	−0.14***	−0.14***	−0.11***	−0.18***	−0.18***	0.02

*CBC, Cognitive-behavioral competence; PI, Positive identity; PA, Prosocial attributes; GPYD, General positive youth development qualities; TPYD, Total positive youth development qualities; LS, Life satisfaction; HL, Hopelessness. ^∗∗∗^p < 0.001.*

**TABLE 3:** Cross-sectional regression analyses.

Predictors		Life satisfaction					Hopelessness				
		*β*	*t*	Cohen’s *f*^2^	*R*^2^ change	F change	*β*	*t*	Cohen’s *f*^2^	*R*^2^ change	F change
**Wave 1**											
	CBC	0.48	33.48***	0.30	0.23	1121.13***	−0.10	−5.97***	0.01	0.01	35.65***
	PA	0.45	30.90***	0.26	0.20	954.50***	−0.11	−0.60.57***	0.01	0.01	43.18***
	PI	0.46	31.42***	0.26	0.21	986.95***	−0.09	−5.55***	0.01	0.01	30.83***
	GPYD	0.59	44.98***	0.54	0.35	2022.73***	−0.16	−9.67***	0.02	0.02	93.56***
	TPYD	0.60	45.87***	0.56	0.36	2104.43***	−0.15	−9.30***	0.02	0.02	86.48***
**Wave 2**											
	CBC	0.44	24.63***	0.24	0.19	606.76***	−0.15	−7.50***	0.02	0.02	56.22***
	PA	0.44	24.57***	0.24	0.19	603.90***	−0.13	−60.91***	0.02	0.02	47.68***
	PI	0.46	26.23***	0.27	0.21	688.05***	−0.11	−5.68***	0.01	0.01	32.29***
	GPYD	0.58	35.74***	0.50	0.33	1276.99***	−0.19	−9.63***	0.04	0.03	92.71***
	TPYD	0.58	35.86***	0.50	0.33	1285.94***	−0.18	−9.46***	0.04	0.03	89.45***

*Control variables were statistically controlled in the first block, and predictors were included in the model separately. CBC, Cognitive-behavioral competence; PI, Positive identity; PA, Prosocial attributes; GPYD, General positive youth development qualities; TPYD, Total positive youth development qualities. ^∗∗∗^p < 0.001.*

**TABLE 4:** Longitudinal regression analyses with separate PYD measures at Wave 1 and life satisfaction and hopelessness at Wave 2.

Predictor		Model 1					Model 2				
		*β*	*t*	Cohen’s *f*^2^	*R*^2^ change	F change	*β*	*t*	Cohen’s *f*^2^	*R*^2^ change	F change
**DV: Life satisfaction (Wave 2)**											
	Life satisfaction (Wave 1)						0.37	20.19***	0.16	0.15	74.69***
	CBC	0.20	10.55***	0.04	0.04	111.37***	0.02	0.98	0.00	0.00	0.95
	PA	0.18	9.24***	0.03	0.03	85.42***	–0.00	–0.05	0.00	0.00	0.00
	PI	0.04	10.53***	0.04	0.04	110.89***	0.03	10.32	0.00	0.00	1.75
	GPYD	0.27	14.32***	0.02	0.08	205.00***	0.07	3.16*	0.00	0.00	10.01
TPYD		0.27	13.92***	0.08	0.07	193.67***	0.06	2.42*	0.00	0.00	5.85*
**DV: Hopelessness (Wave 2)**											
Hopelessness (Wave 1)							0.32	17.05***	0.11	0.12	58.07***
	CBC	–0.18	−9.18***	0.03	0.03	84.28***	–0.15	−8.31***	0.03	0.02	68.97***
	PA	–0.17	−8.93***	0.03	0.03	79.78***	–0.14	−7.73***	0.02	0.02	59.75***
	PI	–0.15	−7.61***	0.02	0.02	57.86***	–0.12	−6.47***	0.02	0.01	41.80***
	GPYD	–0.22	−11.41***	0.05	0.04	130.15***	–0.18	−9.58***	0.04	0.03	91.84***
	TPYD	–0.23	−11.90***	0.05	0.05	141.66***	–0.19	−10.22***	0.04	0.03	104.48***

Control variables were statistically controlled in first block, and predictors were included in the model separately. CBC, Cognitive-behavioral competence; PI, Positive identity; PA, Prosocial attributes; GPYD, General positive youth development qualities; TPYD, Total positive youth development qualities. *p < 0.05; ***p < 0.001.

### Mediation Effects of Life Satisfaction or Hopelessness

The comparison between the two mediation-effect cross-lagged models (see Figure 1) revealed a lower value of AIC (i.e., 3859.01) in the model involving hopelessness as a mediator than that in the competing model involving life satisfaction as a mediator (AIC = 3866.99, see Table 5). The difference in AIC was 7.98, which indicated that the model with hopelessness as the mediator was preferred (Burnham and Anderson, 2002). This model showed adequate model fit χ^2^ = 3713.01, *df* = 333, CFI = 0.92, GFI = 0.91, NNFI = 0.91, RMSEA = 0.062 (90% CI = [0.060, 0.064]) (Kline, 2015). The path coefficients in this model are also shown in Table 5.

**TABLE 5:** Results of cross-lagged mediation models.

Paths	*B*	*SE*	*β*
**Life satisfaction as the mediator only (“path a” only)**			
W1 Positive youth development→W2 Positive youth development	0.48	0.03	0.47***
W1 Life satisfaction→W2 Life satisfaction	0.44	0.04	0.43***
W1 Hopelessness→W2 Hopelessness	0.34	0.02	0.33***
W1 Positive youth development→W2 Life satisfaction	0.07	0.05	0.04
W1 Positive youth development→W2 Hopelessness	–0.35	0.04	−0.18***
W1 Life satisfaction→W2 Positive youth development	0.07	0.02	0.11***
W1 Hopelessness→W2 Positive youth development	–0.01	0.01	–0.02
W2 Life satisfaction→W2 Hopelessness	–0.11	0.03	−0.09***
Model fit:			
χ^2^ = 3720.99, *df* = 333, CFI = 0.92, GFI = 0.91, NNFI = 0.91, RMSEA = 0.062, 90% CI = [0.060, 0.064], AIC = 3866.99			
**Hopelessness as the mediator (“path b” only)**			
W1 Positive youth development→W2 Positive youth development	0.48	0.03	0.46***
W1 Life satisfaction→W2 Life satisfaction	0.44	0.04	0.43***
W1 Hopelessness→W2 Hopelessness	0.34	0.02	0.34***
W1 Positive youth development→W2 Life satisfaction	0.02	0.05	0.01
W1 Positive youth development→W2 Hopelessness	–0.41	0.04	−0.21***
W1 Life satisfaction→W2 Positive youth development	0.07	0.02	0.10***
W1 Hopelessness→W2 Positive youth development	–0.02	0.01	–0.04
W2 Hopelessness →W2 Life satisfaction	–0.08	0.02	−0.10***
Indirect effect of W1 Positive youth development on W2 Life satisfaction via W2 Hopelessness *β* = 0.02*** (SE = 0.01), BC 95% CI = [0.01, 0.04]			
*R*^2^ = 0.30 for W2 Positive youth development			
*R*^2^ = 0.22 for W2 Life satisfaction			
*R*^2^ = 0.19 for W2 Hopelessness			
Model fit:			
χ^2^ = 3713.01, *df* = 333, CFI = 0.92, GFI = 0.91, NNFI = 0.91, RMSEA = 0.062, 90% CI = [0.060, 0.064], AIC = 3859.01			

Control variables (age, gender, and family intactness) were statistically controlled in all models. W1, Wave 1; W2, Wave 2; CI, Confidence interval. ***p < 0.001.

After controlling for the temporal stability of PYD, life satisfaction, and hopelessness in the model, PYD at Wave 1 did not significantly predict life satisfaction at Wave 2 (*β* = 0.02, *p >* 0.05). Alternatively, PYD at Wave 1 showed a significant negative effect on hopelessness at Wave 2 (*β* = –0.21, *p* < 0.001), which subsequently negatively predicted life satisfaction at Wave 2 (*β* = –0.10, *p* < 0.001). Results of bootstrapping showed a significant indirect effect of PYD at Wave 1 on life satisfaction at Wave 2 (*β* = 0.02, *p* < 0.001, BC 95% CI = [0.01, 0.04]) through the mediation effect of hopelessness at Wave 2. Therefore, hopelessness instead of life satisfaction served as a mediator in the present study. In addition, life satisfaction at Wave 1 positively predicted PYD at Wave 2 (*β* = 0.10, *p* < 0.001) while hopelessness at Wave 1 did not significantly predict PYD at Wave 2 (*β* = –0.04, *p* > 0.05) in both models (see Table 5).

As the large sample size in the present study might produce significant findings due to overpowering, we examined the related effect sizes. For the final model, apart from significant temporal effects, the significant standardized regression coefficients included the path from Wave 1 PYD to Wave 2 hopelessness (*β* = –0.21, *p* < 0.001), the path from Wave 1 life satisfaction to Wave 2 PYD (*β* = 0.10, *p* < 0.001), and Wave 2 hopelessness to Wave 2 life satisfaction (*β* = –0.10, *p* < 0.001). The final model explained 30% of the variance in Wave 2 PYD (*R*^2^ = 0.30), 22% of the variance in Wave 2 life satisfaction (*R*^2^ = 0.22), and 19% variance in Wave 2 hopelessness (*R*^2^ = 0.19). Based on Ferguson’s (2009) recommendation (i.e., 0.04 = minimal effect size; 0.25 = moderate effect size), the magnitudes of these *R*^2^ values represented small to moderate effect sizes.

## Discussion

With reference to the research gaps in the scientific literature, there are several innovative features of this study. First, as studies addressing the present research questions did not exist in mainland China, we employed Chinese junior secondary school students in mainland China as participants. Second, in response to the lack of longitudinal studies in this field, two waves of data were collected. Third, to understand the relationship between different aspects of PYD and psychological well-being, we used a validated measure of PYD with 15 primary measures and 4 composite measures assessing different aspects of PYD. Fourth, as few studies have examined PYD attributes and life satisfaction as well as hopelessness simultaneously, both aspects of psychological well-being were included in the present study. Finally, as the role of life satisfaction or hopelessness in the association between PYD attributes and psychological well-being is not conclusive, we explored the mediation effect of life satisfaction versus hopelessness in this study.

Regarding the first two research questions, correlation analyses showed that PYD attributes were concurrently and longitudinally related to life satisfaction and hopelessness. Multiple regression analyses also showed that while Wave 1 PYD attributes positively predicted Wave 2 life satisfaction and its change over time, Wave 1 PYD attributes negatively predicted Wave 2 hopelessness and its change. These findings provide initial support for Hypothesis 1 and Hypothesis 2. The findings are consistent with the general prediction that PYD attributes are positively related to life satisfaction (but negatively related to hopelessness) and they replicate the existing findings in the field (e.g., Valois, 2014). Valois et al. (2009) remarked that “a review of literature yielded few studies exploring the relationship between perceived life satisfaction and developmental assets for adolescents” (p. 317). Similarly, Moksnes et al. (2016) commented that most studies in life satisfaction have “primarily been focusing on adult populations… limited work has examined LS in children and adolescents” (p. 341). As such, the present findings enrich our understanding of the influence of PYD attributes on adolescent psychological well-being.

Regarding Research Question 3, there is stronger support for the hypothesis that PYD attributes were associated with life satisfaction via hopelessness. This observation is novel for two reasons. First, as few related studies have been conducted to look at life satisfaction or hopelessness as a mediator, the present finding is pioneering in nature. Second, this finding is inconsistent with the common conception that PYD attributes would shape subjective well-being (life satisfaction), which would eventually affect psychological ill-being (hopelessness). Nevertheless, it is possible that the lack of PYD attributes among Chinese adolescents leads to a general pessimistic explanatory style and negative experiences of life, which would eventually result in lower life satisfaction. This explanation appears consistent with a few previous findings showing that hopelessness was a significant negative precursor of life satisfaction among high school students (Çapri et al., 2013; Çakar et al., 2015). Of course, as the mediation effect of hopelessness and the outcome (i.e., life satisfaction) took place at Wave 2, the present finding should be interpreted with caution. Additional waves of data should be included in future studies to illuminate the mediation role of life satisfaction or hopelessness.

Another interesting finding in the present study is that life satisfaction showed a positive predictive effect on PYD attributes over time. Previous studies usually treated personal competence such as PYD qualities or internal assets as predictors of life satisfaction. It is nevertheless theoretically plausible that life satisfaction is not just an outcome of different psychological strengths but might also have an influence on the psychological states of an individual. Scholars argued that life satisfaction can serve as an important indicator of psychological state as well as an indicator of PYD (Park, 2004; Moksnes et al., 2016). Several empirical findings give support to this argument: life satisfaction was identified as a mediator on the relationship between friendship quality and social competence (Clayton, 1999); life satisfaction was found to positively predict meaning in life (Heisel and Flett, 2004). A recent study on adolescents in Malaysia revealed that among several predictors of PYD, life satisfaction was the most significant one (Kiadarbandsari, 2015).

In view of the large sample size of the study, the statistically significant findings may be criticized as a result of overpowering. Hence, we examined the effect sizes of the significant paths (Cohen, 1988). Results showed that the effect sizes were in the small to moderate range (Ferguson, 2009). Three points should be taken into account when interpreting these effect size values. First, it is noteworthy that effect sizes in social sciences research are generally small (Ferguson, 2009). In particular, in other cross-lagged panel studies (Martin and Liem, 2010; Raaijmakers et al., 2005; Kobayashi et al., 2020; Wu et al., 2020), small cross-time effects (absolute values of *β* are lower than 0.20, or even lower than 0.10) were commonly reported in leading journals such as *Frontiers in Psychology*. According to Adachi and Willoughby (2015), effect sizes of longitudinal relationships “are dramatically smaller” (p. 116) than those of cross-sectional relationships because controlling stability effect of the outcome (i.e., autoregressive effect) would substantially reduce the effect of a predictor on an outcome over time. Hence, they argued that “longitudinal effect sizes that fall below the universal guidelines for “small” may be incorrectly dismissed as trivial, when they might be meaningful” (p. 116). Besides, researchers suggested that it may be misleading to rigidly follow universal guidelines for interpreting effect sizes in cross-lagged path models that control for stability effects (Peng et al., 2013; Adachi and Willoughby, 2015).

Second, some researchers argued that one should take consistency of findings from different sources and alignment of findings with previous studies and theories when interpreting findings with low effect size. For example, Raaijmakers et al. (2005) reported a significant influence of delinquency on moral reasoning (*β* = −0.06 and −0.07) as well as a significant effect of moral reasoning on delinquency (*β* = −0.08) over time. They argued that such findings are “credible” (p. 256) because the cross-sectional findings and the longitudinal findings are consistent and the findings were in line with other studies and theories. In the same vein, the present findings based on CLPM can be regarded as meaningful because the longitudinal findings are consistent with those based on the related concurrent relationships at Wave 1 and Wave 2. Besides, the findings are consistent with the previous findings (e.g., Valois, 2014) and theoretical predictions of the PYD approach.

The final consideration is that because there were only two waves of data in this study, the readers should interpret the findings with caution. In particular, as pointed out by Fairchild et al. (2009), “although both effect size and mediation have gained attention in recent years, little research has focused on effect-size measures for mediation models” (p. 486). Hence, researchers should collect more waves of data over a longer time span using different types of CLPM in future studies. For example, Hamaker et al. (2015) criticized the traditional CLPM approach and proposed the random intercepts cross-lagged panel models (RI-CLPM) for studies with three or more waves of data. Nevertheless, as there are different approaches of data analyses, they also emphasized that “without a doubt, there will be many instances where another approach is more suited” (p. 112).

Another observation is that compared to the previous findings that the magnitude of 1-year autoregressive coefficients for PYD and well-being constructs was around or higher than 0.60 (Yu and Shek, 2018; James and Ward, 2019), the temporal stability of PYD and well-being measures was not high (ranging between 0.34 and 0.48) in the present study. The relatively “lower” autoregressive coefficients in the present study can be explained by the fact that PYD, life satisfaction, and hopelessness are not stable trait-like constructs because they keep on developing during adolescence (Hamaker et al., 2015). In particular, the core belief of PYD approach is developmental plasticity which emphasizes the possibility of change in adolescents. In addition, it is not uncommon to identify relatively lower autoregressive effects in cross-lagged models involving PYD and well-being factors. For example, Shek and Zhu (2019) reported a 1-year autoregressive coefficient around 0.40 for moral competence; Yu and Shek (2018) reported 1-year autoregressive coefficients ranging between 0.41 and 0.55 for hopelessness; and James and Ward (2019) reported a 1-year autoregressive coefficient of 0.18 for perceived spirituality. Similarly, the 2-year autoregressive coefficients of PYD-related constructs, such as self-efficacy and self-regulation, were around 0.30 in recent studies (Tovel et al., 2019; Memmott-Elison et al., 2020).

In addition to the above theoretical contributions to the scientific literature on PYD, our findings replicated the previous Western findings and showed that PYD attributes are positively related to adolescent psychological well-being in a non-Western context. Hence, it can be reasoned that the assertions of this Western PYD model (e.g., PYD attributes promote psychological well-being) and related findings are generalizable to Chinese adolescents in mainland China (i.e., generalizability across people and places). In conjunction with findings reported in other cultural contexts, the present findings suggest that there is support for the relationship between PYD attributes and adolescent well-being in different cultural contexts. However, we have to consolidate and replicate the present findings in the Chinese context in future. Nevertheless, the present findings are important because research in developmental psychology has been criticized as “WEIRD” where participants from “Western, educated, industrial, rich and democratic” societies have been commonly recruited (Nielsen et al., 2017). The resemblance of the present findings to Western findings suggests that the benefit of PYD attributes on adolescent psychological well-being exists in different cultural contexts.

Obviously, one should ask why the related Western findings are replicated and why the assertions in the PYD model proposed by Catalano et al. (2004) are supported in the present study. There are two possible explanations for this observation. The first explanation is that with rapid modernization and Westernization in mainland China in the past four decades, Chinese people have become more Westernized and they share Western individualistic values (Sun and Wang, 2010; Steele and Lynch, 2013; Cai et al., 2018). The second explanation is that some of the Western PYD attributes are also emphasized in the Chinese culture. For example, Chinese people emphasize the importance of family support which constitutes “bonding” for adolescents. Chinese people’s emphasis on moral character and law abidance is in line with the PYD attributes of “moral competence” and “upholding prosocial norms.” In fact, the principle of “*gu ben pei yuan*” (i.e., one does not get sick if the inner energy is consolidated) in Chinese medicine is consistent with the PYD notion that inner developmental assets such as character contribute to positive adolescent development. Conceptually speaking, it would be interesting to examine how PYD attributes are linked to Chinese concepts on adolescent development. Actually, there are views suggesting that some of the PYD attributes are consistent with some indigenous Chinese concepts on adolescent development. For example, Shek et al. (2013) showed that some Confucian virtues are conceptually similar to concepts related to character strengths and PYD attributes. Shek and Law (2019) also pointed out some of the emphases in *Di Zi Gui* are consistent with the principles in modern parenting science.

Practically, the present findings reinforce the thesis that the promotion of PYD attributes in adolescents can help to promote their mental health. In view of the rising adolescent mental problems in the global context (e.g., World Health Organization, 2019), researchers, practitioners, and policymakers are finding ways to prevent adolescent mental health problems. With specific reference to the Chinese cultural context, Mei et al. (2019) reported that Chinese adolescents face a high academic learning pressure because the education system in China morbidly emphasizes the significance of academic achievement. Based on surveys involving 50,361 primary and junior secondary school students in mainland China, Liu et al. (2018) identified that 54% of the respondents were at risk of having at least one type of anxiety and learning anxiety emerged as the most prevalent anxiety risk that affected 47% of the students. Hence, there is a need to explore how we can protect the mental health of mainland Chinese students.

Obviously, PYD is an evidence-based approach to help young people to build up their inner strengths so that they can be protected from risky behavior and thrive. Unfortunately, although there are many PYD programs in Western contexts, PYD programs in Chinese communities are sparse. In Hong Kong, a PYD program entitled “Project P.A.T.H.S.” has been developed and rigorously evaluated. Evaluation findings based on different strategies showed that the project protected young people from risk behavior and promoted their thriving (e.g., Catalano et al., 2012; World Health Organization, 2016; Alvarado et al., 2017). In view of the very positive evaluation of the “Project P.A.T.H.S.” in Hong Kong, Tin Ka Ping Foundation provided financial support to transplant the program to mainland China (i.e., “Tin Ka Ping P.A.T.H.S. Project”) where the program has displayed very good impacts as well (Shek, 2019; Shek et al., 2019b; Zhu and Shek, 2020). As there are growing mental health problems among Chinese adolescents (e.g., Lo et al., 2018; Shek and Siu, 2019), PYD programs constitute a promising approach to promote adolescent holistic development utilizing the principles of primary prevention.

Several limitations of the study should be noted. First, as there were only two waves of data, collecting more waves of data would be necessary. Second, as mediating factors and outcomes were based on Wave 2 data, the findings have obvious limitation. Third, as only schools from four cities in China were recruited, the present study should be replicated in other places of China. Fourth, as only self-report data from adolescents were employed, additional data collected from other stakeholders such as the parents and teachers can portray a richer picture on the problem area. Fifth, apart from life satisfaction and hopelessness, it would be illuminating if more measures of subjective well-being and psychological ill-being could be employed in future studies. Sixth, although CPYDS measuring PYD attributes is locally developed, scales assessing life satisfaction and hopelessness are translated and adapted measures. Although these two scales also showed good psychometric properties, using indigenously developed measures of psychological well-being would be helpful in future. Finally, in view of the complex inter-relationships between PYD attributes and different aspects of psychological well-being, more work should be conducted.

## Data Availability Statement

The raw data supporting the conclusions of this article will be made available by the authors, without undue reservation, to any qualified researcher.

## Ethics Statement

The studies involving human participants were reviewed and approved by The Hong Kong Polytechnic University. Written informed consent to participate in this study was provided by the participants’ legal guardian/next of kin.

## Author Contributions

ZZ contributed to the writing and polishing of the manuscript, conducted literature review on the related studies in mainland China and wrote related parts, and checked the accuracy of the content and analyses. DS conceived the project, drafted the original version of the manuscript, and edited the final version of the manuscript. XZ contributed to data collection and analyses as well as drafting the writing on the methodology and results of the study. All authors contributed to the article and approved the submitted version.

## Conflict of Interest

The authors declare that the research was conducted in the absence of any commercial or financial relationships that could be construed as a potential conflict of interest.
